# Commencement of and Retention in Web-Based Interventions and Response to Prompts and Reminders: Longitudinal Observational Study Based on Two Randomized Controlled Trials

**DOI:** 10.2196/24590

**Published:** 2021-03-12

**Authors:** Athanasios Andriopoulos, Erik M G Olsson, Ylva Hägg Sylvén, Jonas Sjöström, Birgitta Johansson, Louise von Essen, Helena Grönqvist

**Affiliations:** 1 Department of Women's and Children's Health Uppsala University Uppsala Sweden; 2 Department of Informatics and Media Uppsala University Visby Sweden; 3 Immunology, Genetics and Pathology Uppsala University Uppsala Sweden

**Keywords:** log data analysis, use pattern, retention, dropout, attrition, online intervention, online data

## Abstract

**Background:**

Web-based interventions are effective for several psychological problems. However, recruitment, adherence, and missing data are challenges when evaluating these interventions.

**Objective:**

This study aimed to describe the use patterns during the commencement phase, possible retention patterns (continuation of data provision), and responses to prompts and reminders among participants in 2 randomized controlled trials (RCTs) evaluating web-based interventions.

**Methods:**

Data on use patterns logged in 2 RCTs aiming to reduce symptoms of anxiety and depression among adult patients recently diagnosed with cancer (AdultCan RCT) and patients with a recent myocardial infarction (Heart RCT) were analyzed. The web-based intervention in the AdultCan trial consisted of unguided self-help and psychoeducation and that in the Heart trial consisted of therapist-supported cognitive behavioral therapy. In total, 2360 participants’ use patterns at first log-in, including data collection at baseline (ie, commencement) and at 2 follow-ups, were analyzed. Both the intervention and comparison groups were analyzed.

**Results:**

At commencement, 70.85% (909/1283) and 86.82% (935/1077) of the participants in AdultCan and Heart RCTs, respectively, logged in and completed baseline data collection after receiving a welcome email with log-in credentials. The median duration of the first log-in was 44 minutes and 38 minutes in AdultCan and Heart RCTs, respectively. Slightly less than half of the participants’ first log-ins were completed outside standard office hours. More than 80% (92/114 and 103/111) of the participants in both trials explored the intervention within 2 weeks of being randomized to the treatment group, with a median duration of 7 minutes and 47 minutes in AdultCan and Heart RCTs, respectively. There was a significant association between intervention exploration time during the first 2 weeks and retention in the Heart trial but not in the AdultCan trial. However, the control group was most likely to retain and provide complete follow-up data. Across the 3 time points of data collection explored in this study, the proportion of participants responding to all questionnaires within 1 week from the prompt, without a reminder, varied between 35.45% (413/1165) and 66.3% (112/169). After 2 reminders, up to 97.6% (165/169) of the participants responded.

**Conclusions:**

Most participants in both RCTs completed the baseline questionnaires within 1 week of receiving the welcome email. Approximately half of them answered questions at baseline data collection outside office hours, suggesting that the time flexibility inherent in web-based interventions contributes to commencement and use. In contrast to what was expected, the intervention groups generally had lower completion rates than the comparison groups. About half of the participants completed the questionnaires without a reminder, but thereafter, reminders contributed to both baseline and follow-up retention, suggesting they were effective. Strategies to increase commencement of and retention in eHealth interventions are important for the future development of effective interventions and relevant research.

## Introduction

### Background

Web-based interventions are efficient for mental health problems such as symptoms of anxiety, depression, and posttraumatic stress [1-4], with effects lasting up to 3 years after treatment [5-7]. However, studies evaluating web-based interventions struggle with low use, where some participants never log in or commence the intervention at all [8,9] and where retention rates, that is, the continuation of participant data provision, vary between 17% and 98% [10-13]. The problem of low retention has been continuously reported in relation to web-based interventions and research and has even been discussed in terms of *the law of attrition* [13]. Although this problem is not unique to eHealth, the complexity of the field makes attrition almost inevitable, and it is thus important to highlight, measure, and discuss its determinants to be able to improve future eHealth interventions and research, for example, regarding usability, efficacy, and increased acceptability [13]. Disease severity [14], symptoms of anxiety [15], technical issues, lack of motivation, time constraints, the complexity of the intervention, low expectations of its efficacy, compatibility with participants’ profiles, and current needs [13,16] have been reported by participants as reasons for noncommencement and low retention [8,17-21]. Demographic variables such as younger age, higher level of education, and female gender are often associated with increased retention in web-based intervention studies [22].

In web-based intervention trials, it is possible to track participants’ activities in the intervention by logging use patterns with high precision, including recording every click a participant makes on the web-based platform when working with the intervention and answering questionnaires [23]. Automatized and standardized reminders via emails or text messages are often used to support retention at low cost and with minimal effort [12]. Previous findings indicate that the majority of participants in randomized controlled trials (RCTs) evaluating a web-based intervention perceived reminders as harmless, well accepted, and useful, but the effectiveness of reminders in increasing retention in this type of intervention has seldom been evaluated systematically [24,25]. Log data could be valuable for analyzing the patients’ use patterns in web-based interventions and their overall utility [26,27].

### Aim and Research Questions

The overall aim of this study is to describe use patterns of participants in 2 RCTs evaluating web-based interventions aimed at reducing symptoms of anxiety and depression in adult patients with cancer (AdultCan trial [28]) and patients who recently had a myocardial infarction (Heart trial [8]) when (1) logging in to the portal for the first time for completing baseline questionnaires (ie, commencement), (2) completing questionnaires at the first and second follow-ups (ie, retention), and (3) responding to prompts and reminders to fill in questionnaires (ie, responses).

#### Research Questions Regarding Commencement

The research questions regarding commencement were as follows:

- How many potential participants completed the baseline questionnaires and how many left it incomplete?
- Was there a difference in sex or age between those who completed the baseline questionnaires and those who did not?
- How many days after invitation did the participants complete the baseline questionnaires and how many logged in more than once before completing them?
- How long did it take to complete the baseline questionnaires and at what time of the day were the questionnaires completed?

#### Research Questions Regarding Retention

The research questions regarding retention were as follows:

- How long did the participants explore the intervention after randomization and how many completed follow-up 1 and follow-up 2?
- Was there a difference between those allocated to treatment versus those not regarding completion of follow-up questionnaires?
- Was there an association between exploring activity during the first 2 weeks and completion of follow-up questionnaires?

#### Research Questions Regarding Prompts and Reminders

The research questions regarding prompts and reminders were as follows:

- How many participants completed questionnaires at follow-up 1 and 2, respectively, after being prompted to do so, and how many responded to questionnaires at follow-ups 1 and 2, respectively, after being reminded one or two times?

## Methods

### Design

The study had a longitudinal and descriptive correlational design and used secondary data analysis. The primary analysis of the efficacy of the interventions has been reported elsewhere [8,28].

### Setting

The Uppsala University Psychosocial Care (U-CARE) program has the overarching goal of promoting psychosocial health among patients struck by somatic diseases and their significant others [23].

The 2 RCTs explored in this study, AdultCan [28] and Heart [8], were conducted via the U-CARE portal (hereafter, portal), a secure web portal developed within U-CARE.

In the AdultCan trial, a stepped-care (consisting of 2 steps) web-based intervention was evaluated. The first step, available for 24 months for each participant, consisted of information, psychoeducation, and self-help material including texts, video lectures, discussion forums, and the possibility for participants to ask questions about cancer and its treatment and get answers from experts. Participants still reported anxiety and depression after access to the first step, and after 1, 4, or 7 months, they were offered a second step consisting of 10 weeks of therapist-supported internet-based cognitive behavioral therapy (iCBT) [28]. Log data collected via the portal during the first step of the intervention were analyzed in this study.

In the Heart trial, a web-based intervention consisting of 14 weeks of therapist-supported iCBT, including self-help material, homework assignments, web-based contact with a therapist, and peer support via a discussion forum, was evaluated. The intervention included 10 modules, for example, behavioral activation, cognitive restructuring, exposure, and problem solving. Participants could choose which modules to work with and receive weekly therapist support [8].

### Participants

Log data from 1283 participants in the AdultCan trial and 1077 participants in the Heart trial were analyzed. In the AdultCan trial, the inclusion criteria were patients with newly (within 6 months) diagnosed breast, prostate, or colorectal cancer as well as patients with recurrence of colorectal cancer (within 6 months of diagnosis) at 3 hospitals in Sweden. Exclusion criteria were inability to read and understand Swedish, cognitive disability (eg, dementia or psychosis), a constant need for care (Karnofsky score<40), short expected survival (<3 months), severe depression or suicide risk with regard to answers on the Montgomery-Åsberg Depression Rating Scale-Self-Report (MADRS-S) measure, and participation in a competing clinical trial including prostate cancer patients receiving radiotherapy.

In the Heart trial, inclusion criteria were >7 on one or both of the Hospital Anxiety and Depression Scale (HADS) subscales. Exclusion criteria were scheduled for coronary artery bypass surgery; inability to use a computer, internet, email, or mobile phone; unable to read Swedish; expected to live <1 year; anticipated to show poor compliance (eg, substance abuse); self-reported severe depression or suicidal ideation; MADRS-S item 9>3; and participation in another behavioral intervention trial. Detailed information about the methods used in the AdultCan and Heart RCTs is provided elsewhere [8,28]. In this study, participants who provided informed consent and were added to the portal were considered as participants.

### Procedure

In both studies, participants who self-reported symptoms of anxiety and/or depression above the cut-off >7 on any of the subscales of the HADS were randomized to either the treatment group or the control group. In the AdultCan trial, those scoring below the cut-off on both subscales were assigned to a reference group that was followed longitudinally. Details of the procedure at commencement and data collection in the AdultCan and Heart trials are presented in Table 1.

**Table 1:** The procedure at commencement and follow-up data collection in AdultCan and Heart trials.

Phase, Studies		
	AdultCan trial	Heart trial
**Commencement**		
	Eligible persons were informed about the study at a regular hospital visit or by telephone within 6 months after being diagnosed with cancer. After providing written informed consent, participants received a welcome email with log-in credentials to the portal for baseline questionnaires. Participants were informed that if all baseline questions were not answered within 24 h, they would have to restart from scratch. If participants did not complete the 14 baseline questionnaires within 7 days, they received a reminder via SMS and email. If participants did not complete the baseline questionnaires within 14 days, they received a second reminder via SMS and email. If participants had still not completed the baseline questionnaires 30 days after the prompt, study personnel contacted them, if possible, by telephone and reminded them to respond to the questionnaires. Participants scoring above the cut-off on HADS^a^ were randomized to the treatment or control group in the portal. The log-in session where a participant is randomized is called the “randomization session.” Participants scoring below the cut-off were assigned to the reference group and were asked to answer questionnaires at selected time points. Participants randomized to the treatment group got immediate access to the first step of the intervention via the portal.	Eligible persons were informed about the study at a regular hospital visit shortly after discharge from the hospital after an MI^b^. Potential participants were then contacted again 8 weeks after the MI by the study staff via telephone. After providing written informed consent, participants received a welcome email with log-in credentials to the portal for baseline questionnaires. Participants were informed that if all baseline questions were not answered within 24 h, they would have to restart from scratch. If participants did not complete the 13 baseline questionnaires within 7 days, they received a reminder via SMS and email. If participants did not complete the baseline questionnaires within 14 days, they were reminded by study personnel via telephone. Participants scoring above the cut-off on HADS were randomized to the treatment or control group in the portal. The log-in session where a participant is randomized is called the “randomization session.” Participants randomized to the treatment group got immediate access to the intervention via the portal.
**Retention, prompts, and reminders**		
	Follow-up 1: 2 weeks after randomization, participants were asked to complete 1 (control group) or 2 (treatment group) questionnaires. Follow-up 2: 1 month after randomization, participants were asked to complete 4 (reference group), 8 (control group), or 10 (treatment group) questionnaires. At follow-up 1 and 2 participants were prompted via email and SMS to log in and complete the questionnaires. If participants did not complete the questionnaires within 7 days after the prompt, they received a first reminder via SMS and email. If participants did not complete the questionnaires within 12 days after the prompt, they received a second reminder via SMS and email.	Follow-up 1: 5 weeks after randomization, participants were asked to complete 4 questionnaires. Follow-up 2: 14 weeks after randomization, participants were asked to complete 14 questionnaires. At follow-up 1 and 2 participants were prompted via email and SMS to log in and complete questionnaires. If participants did not complete the questionnaires within 7 days after the prompt, they received a first reminder via SMS and email. If participants did not complete the questionnaires within 14 days after the prompt, study personnel contacted them via telephone and reminded them to respond to the questionnaires.

^a^HADS: Hospital Anxiety and Depression Scale.

^b^MI: myocardial infarction.

Participants logged in to the portal with double authentication, entering username, personal password, and a temporary 5-digit code that they received in an SMS. A log-in session ended when a participant logged out of the portal or was inactive for more than 20 minutes.

In the Heart trial, at the second reminder, participants were offered to use paper forms to answer questionnaires at follow-up 1 and 2, which 27 and 46 participants did at the 2 follow-ups, respectively. Thus, the web-based completion rate is only a part of the total completion rate in the Heart trial.

### Data and Data Collection

Log data from the full duration of the AdultCan and Heart trials were collected from April 16, 2013, to April 28, 2017, and were exported from the portal by a system developer. The data were reviewed by a second system developer. In addition, the researchers performed random checks and reviewed any inconsistencies.

Log data refers to records of real-time actions performed by each user, and mouse clicks and keyboard strokes are logged as user actions with time stamps. In this study, log data at commencement, during the 2-week period following commencement, and at 2 consecutive follow-up time points within the RCTs AdultCan [28] and Heart [8] were collected via the secure portal developed within U-CARE.

#### Variables

The variables used to answer the research questions are presented in Table 2.

**Table 2:** Variables used in the study.

Phase and study variables measured		Value
**Commencement**		
	Participant commencing answering questionnaires at baseline	y/n^a^
	Participant completing answering questionnaires at baseline	y/n
	Time from the welcome email sent from the portal with log-in credentials to participant’s first log-in	d^b^;h:min
	Duration of participant first log-in	min:s
	Time of the day when the participant first logged in	h:min
	Day of week when the participant first logged in	Mo-Su^c^
	Whether the participant’s first log-in ended with a click by the participant on the log-out button or if the participant was automatically logged out after being inactive (passive log-out)	y/n
**Retention**		
	Participant explored the intervention in randomization session (treatment group only)	y/n
	Participant explored the intervention within 14 days after randomization (treatment group only)	y/n
	Length of time the participant explored the intervention within 14 days after randomization (treatment group only)	min:s
	Participant completed all questionnaires at follow-up 1 and 2	y/n
**Response to prompts and reminders**		
	Number of prompts and/or reminders sent to participants at the 3 data collection time points	0-2

^a^y/n: yes or no.

^b^d: day.

^c^Mo-Su: Monday to Sunday.

The following portal activities were defined as exploring the intervention: any click in the library, forum, chat, diary, FAQ, ask an expert, using the internal message system, and the iCBT program.

Self-reported demographical data were collected at baseline.

#### Missing Data

Missing data were mostly because messages, such as prompts and reminders, from the portal were not logged properly, as a result of a temporary technical error in the early phase after launching the studies. The welcome emails with log-in credentials were erroneously logged for 5 and 7 participants in the respective studies, and reminders to log in to the portal to answer questionnaires at baseline were erroneously or insufficiently logged for 118 and 50 participants from the AdultCan and Heart RCTs, respectively, with missing data as a result. The corresponding figures for the first follow-up were 76 and 19, and for the second follow-up, 112 and 18. In the Heart trial, 68, 24, and 42 participants were not reached by telephone for reminders at baseline, first, and second follow-up, respectively. The country of birth was not reported by one participant. When investigating exploration, 10 participants in the AdultCan trial and 7 in the Heart trial had missing data.

### Statistical Analysis

Descriptive statistics were used to examine and report all variables. Medians were used when the frequency distributions were skewed. Pearson chi-square test was used to examine potential differences between the numbers of participants exploring the intervention among participants who completed the baseline (completers) and those who did not complete the baseline (noncompleters) in the respective study groups. The Mann-Whitney U-test was used to examine potential associations between time used to explore the intervention and if participants completed the data collections in the respective studies. Actual *P* values are reported. All analyses were based on complete data, that is, no imputations were performed.

Data were analyzed using IBM SPSS Statistics V25.0 and STATA v 15.1.

## Results

### Patient Characteristics

Participants in the AdultCan and Heart trials who completed baseline questionnaires had a mean age of 61 years (SD 10.6) and 62 years (SD 8.1), respectively, and at least 90.29% (1665/1844) were born in Sweden, and more than 44.74% (825/1844) had some university education. In the AdultCan trial, the proportion of female participants was more than double that in the Heart trial (for more details, Table 3).

**Table 3:** Characteristics and commencement data of participants.

Characteristics and commencement data		AdultCan trial				Heart trial			
		Completed BL questionnaire^a^ (n=909)		Did not complete BL questionnaire(n=374)		Completed BL questionnaire(n=935)		Did not complete BL questionnaire(n=142)	
Age (years), mean (SD)		61.3 (11)		62.5 (11)		62.2 (8)		62.4 (9)	
Women, n (%)		525 (57.8)		207 (55.3)		220 (23.5)		34 (23.9)	
Born in Sweden, n (%)		819 (90.1)		N/A^b^		846 (90.5)		N/A	
**Highest level of education, n (%)**									
	Elementary school		184 (20.2)		N/A		190 (20.3)		N/A
	High school		296 (32.6)		N/A		349 (37.3)		N/A
	University ≤3 years		193 (21.2)		N/A		185 (29.8)		N/A
	University >3 years		236 (26.0)		N/A		211 (22.6)		N/A
**Study group,** **n** **(%)**									
	Reference group		664 (72.9)		N/A		696 (74.4)		N/A
	Control group		121 (13.3)		N/A		122 (13.0)		N/A
	Treatment group		124 (13.6)		N/A		117 (12.5)		N/A
**Log-ins for noncompleters BL questionnaire,** **n** **(%)**									
	≥1 log-ins		N/A		83 (22.2)		N/A		33 (23.2)
	No log-ins		N/A		291 (77.8)		N/A		109 (76.8)
Time to first log-in (d;h:min), median (range)		6;11:59 (0;00:01-59;04:44)		N/A		3;21:57 (0;00:02-18;10:32)		N/A	
First log-in duration (min:s), median (range)		44:08 (00:31-180:06)		N/A		37:43 (00:19-326:48)		N/A	

^a^BL: baseline.

^b^N/A: not applicable

### Commencement

A total of 70.85% (909/1283) of participants in the AdultCan trial and 86.82% (935/1077) of participants in the Heart trial completed all questionnaires at baseline (Table 3). Moreover, 6.47% (83/1283) of the participants in the AdultCan trial did not complete all baseline questionnaires after being logged in at least once, and 22.68% (291/1283) of participants did not log in at all. In the Heart trial, these numbers were approximately half (Table 3). The median response time, from receiving the welcome email to the first log-in, was slightly more than 6 days in the AdultCan trial and almost 4 days in the Heart trial. Of those who completed all questionnaires at baseline, 24.9% (226/909) in the AdultCan trial and 23.7% (222/935) in the Heart trial logged in more than once before completing the baseline questionnaires. Of those who completed all questionnaires at baseline, 73.0% (664/909) in the AdultCan trial and 74.4% (696/935) in the Heart trial were allocated to the reference group and not randomized. The median duration of the first log-in, from the first to the last click, was 44 minutes and 38 minutes for the AdultCan and Heart trials, respectively. At baseline, 54.3% (494/909) of the participants in the AdultCan trial and 35.8% (335/935)of the participants in the Heart trial logged out of the portal by a click. No differences in age or gender were found between participants who completed the baseline questionnaires and those who did not.

**Figure 1:**
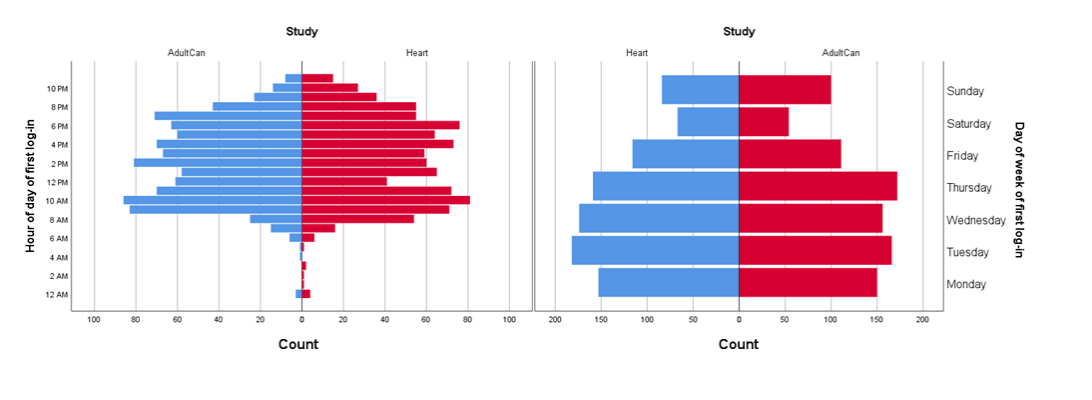
The time of day and day of week for participants’ first log-in.

The times when the participants logged in for the first time are illustrated in Figure 1. In the AdultCan trial, 54.8% (498/909) of the first-time log-ins were on weekdays between 8 AM and 5 PM, representing normal office hours. The corresponding figure for the Heart trial was 52.2% (488/935).

### Retention

**Figure 2:**
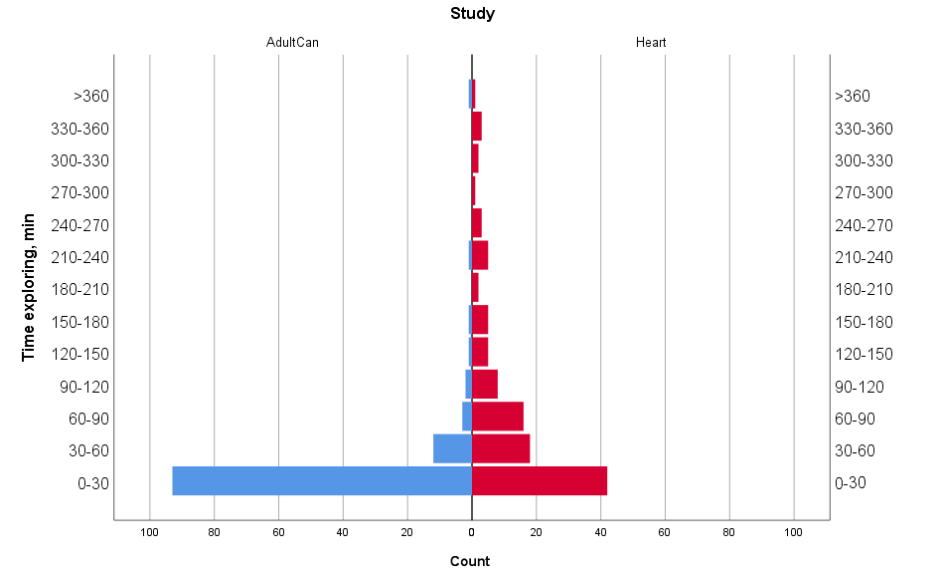
Total time spent in exploring the intervention within 14 days after randomization by number of participants in the treatment group in AdultCan and Heart trials.

A total of 73.7% (84/114) of those randomized to treatment in the AdultCan trial and 70.3% (78/111) of those in the Heart trial explored the intervention within the session when they completed baseline questionnaires and were randomized to the treatment group. Thereafter, within a 14 day-period after randomization, separate from the randomization session, 29.8% (34/114) of the participants in the AdultCan trial and 72.1% (80/111) of the participants in the Heart trial explored the intervention at least once. The median total time participants were exploring the intervention during the first 14 days after randomization was 7 minutes for the AdultCan trial and 47 minutes for the Heart trial (Table 4). Figure 2 provides a detailed description of the distribution of total time spent exploring the intervention.

**Table 4:** Number of participants in the treatment group exploring the intervention within 14 days after randomization and their time spent on exploring.

Measures of exploration	AdultCan trial (n=144)	Heart trial (n=111)
Exploring the intervention, n (%)	92 (80.7)	103 (92.8)
Total time spent exploring the intervention (min:s), median (range)	6:58 (00:01-461:40)	47:14 (00:22-486:06)

Overall, the questionnaires were completed using the web-based platform by 44%-85% of the participants in the different study groups (treatment, control, and reference group) at the 2 follow-ups (Table 5). In the Heart trial, more participants in the control group compared with the treatment group completed the questionnaires using the web-based platform both at follow-up 1 and follow-up 2. No differences between the groups were found in the AdultCan trial at follow-up 1, but at follow-up 2, the reference group completed the questionnaires the most and the treatment group the least (Table 5).

**Table 5:** Web-based completion rates for the follow-ups for the AdultCan and Heart trials.

Follow-up completion	AdultCan trial					Heart trial		
	Reference, n (%)	Control (n=121), n (%)	Treatment (n=124), n (%)	*P* value^a^	Control (n=121), n (%)		Treatment (n=117), n (%)	*P* value^a^
Completing FU^b^1	N/A^c^	82 (67.7)	82 (66.1)	.79	104 (85.2)		80 (68.3)	.002
Completing FU2	558 (84)	94 (77.7)	90 (72.6)	.005	96 (78.7)		52 (44.4)	<.001

^a^*P* values from chi-square tests.

^b^FU: follow-up.

^c^N/A: not applicable.

When combining total exploration time, the first 14 days with retention, completing follow-up 1 and follow-up 2 were positively associated with the exploration time for the first 14 days after randomization in the Heart trial but not with any of the follow-ups in the AdultCan trial (Table 6). When dividing participants in the Heart trial into an active and a passive treatment group, based on a median split in exploration time (median 47 minutes and 14 seconds), 55% (30/55) in the passive treatment group compared with 80% (45/56) in the active group and 85.2% (104/122) in the control group completed follow-up 1. At follow-up 2, the corresponding figures were 31% (17/55), 55% (31/56), and 62.3% (96/122).

**Table 6:** Total time exploring the intervention during the first 14 days cross-tabled with completion of follow-up measures.

Follow-up		AdultCan trial (n=114)				Heart trial (n=111)			
		Completed web-based follow-up	Did not complete web-based follow-up	Diff.^a^	*P* value^b^	Completed using web-based intervention	Did not complete web-based follow-up	Diff.	*P* value^b^
**Follow-up 1**									
	n (%)	75 (66)	39 (34)			75 (68)	36 (32)		
	Total time spent exploring the intervention (min:s), median (range)	6:35 (00:01-461:40)	07:20 (00:01-76:05)	−0:45	.55	70:37 (00:23-353:40)	23:53 (00:22-486:05)	46:44	<.001
**Follow-up 2**									
	n (%)	83 (73)	31 (27)			48 (43)	63 (57)		
	Total time spent exploring the intervention (min:s), median (range)	7:34 (00:01-461:40)	4:08 (00:02-75:05)	3:26	.46	76:16 (00:22-353:40)	34:03 (00:23-486:05)	42:13	.005

^a^Diff.: difference in median times between those who completed web-based follow-ups and those who did not.

^b^*P* values from Mann-Whitney U-tests.

### Response to Prompts and Reminders

Across the 3 data collection time points explored in this study, the proportion of participants responding within 7 days from the prompt without a reminder was between 36% and 66%. Within 5 days of the first reminder, sent out via SMS and email, an additional 40%-86% of the remaining participants responded. In the AdultCan trial, the second reminder, sent via SMS and email, generated between 36% and 50% additional responses from the remaining participants. In the Heart trial, the second reminder, via telephone, generated 34%-69% additional responses among those who had not responded so far (Table 7).

**Table 7:** Responses to reminders at baseline and the 2 follow-ups.

Time of response	AdultCan						Heart					
	Baseline		FU^a^1		FU2		Baseline		FU1		FU2	
	N	n (%)	N	n (%)	N	n (%)	N	n (%)	N	n (%)	N	n (%)
Response after prompt, n (% of all)	1165	413 (35.5)	169	112 (66.2)	797	442 (55.5)	959	550 (57.3)	196	109 (64.5)	179	74 (41.3)
Response to the first reminder, n (% of remaining)	752	299 (39.8)	57	49 (86.0)	355	151 (42.5)	409	209 (51.1)	87	50 (57.5)	105	44 (41.9)
Response to the second reminder, n (% of remaining)	453	162 (35.8)	8	4 (50.0)	204	99 (48.5)	200	137 (68.5)	37	16 (43.2)	61	23 (37.7)
No response, n (% of all)	1165	291 (25.0)	169	4 (2.4)	797	105 (13.2)	959	63 (6.6)	196	21 (10.7)	179	38 (21.2)

^a^FU: follow-up.

## Discussion

### Principal Findings

The results show that at commencement, most recruited and consenting participants logged in and completed the baseline questionnaires. Most nonresponders did not log in at all. In contrast to previous studies [29] that have indicated that age and gender are related to attrition, no difference in gender or age was found between the participants who finished baseline and those who did not. This may be partly because of age heterogeneity. Fewer participants in the AdultCan trial than those in the Heart trial completed the baseline questionnaires. This could have many reasons, such as recruitment procedure, intervention type, disease severity, and so on. Most participants in the AdultCan trial were undergoing active cancer treatment at the time of inclusion, whereas the focus of participants in the Heart trial was on secondary prevention. Most participants who completed the baseline questionnaires completed the questionnaire within 1 week of receiving the welcome email with log-in credentials from the portal.

One argument for using web-based interventions is that they can be accessed at any time. Although most participants had their first log-in on weekdays and during the day, 45%-48% of the participants chose to commence the studies outside common office hours when face-to-face psychological support is usually not offered. The log-in times were similar in the 2 studies regarding time of day and day of the week and also similar to what has been reported in other studies [30]. It is known that the time of day and the day of week people prefer to answer surveys are related to sociodemographic and health characteristics [31]. As internet interventions are flexible in time, they may be able to reach patients in need at convenient times.

Most participants opened at least one item of the intervention directly after being randomized to treatment. Furthermore, in the Heart trial, 72% of the participants explored the intervention in separate sessions during the following 14 days. This was more than that in the AdultCan trial. Median time logged in during the first 14 days was also longer in the Heart trial than in the AdultCan trial. This was expected owing to the intervention formats, as the Heart trial was a therapist-supported iCBT intervention, whereas the AdultCan trial offered self-help psychoeducation without individual support during the first 2 weeks examined in this study. In addition, therapist-supported iCBT was restricted to 10 weeks, whereas self-help psychoeducation in the AdultCan trial was available for 24 months. However, the overall intervention use over the first 2 weeks was relatively low. Persuasive features such as feedback have been suggested to increase use [32] and were available in the Heart intervention. However, the participants had to log in without any specific prompts to notice the feedback.

Most participants (66%-85%) were retained in the studies and answered the follow-up questionnaires. When comparing completion rates between the study groups, the control group in the Heart trial had a higher rate than that in the treatment group at both follow-ups. A similar pattern was evident in the AdultCan trial at follow-up 2, where the reference group had the highest completion rate and the treatment group the lowest. Although the more active treatment participants in the Heart study also had a higher completion rate than those who were less active, the active participants were still less likely to complete the follow-ups than the control group. This was an unexpected finding. It may be that participants felt obliged to contribute to a certain amount and that those participating in the intervention thought they had filled their quota even before the follow-up questionnaires. To the best of our knowledge, there are no previous systematically summarized studies reflecting on such patterns.

Prompts and reminders for completing questionnaires were sent via SMS and email. Most participants answered the questionnaires after the prompt without any reminders. However, the following 2 consecutive reminders were useful in increasing the response rates, not only when executed via telephone calls but also via SMS and email. The results are in line with previous research showing that reminders contribute to the overall response rate [33] and that participants find reminders acceptable and useful [25]. In the Heart trial, participants were offered paper forms as a secondary response alternative at the second reminder, which should be considered when interpreting the sometimes very low retention rates.

### Strengths and Limitations

The log data collected for this study allowed for a unique possibility of exploring these aspects that are important for the success of web-based interventions. Using participants in 2 web-based intervention studies gave us a large sample size of 2360 participants. There are several differences between the studies, making them difficult to compare; hence, they are described as separate cases with few comparisons. However, the results were similar, and the 2 cases provided cumulative information for the exploration of use patterns. Another strength is that both studies recruited clinically and consecutively, resulting in a sample from all patients, not only self-selected highly motivated participants in web-based interventions. We believe that a more detailed log data on participants’ use patterns could improve the development of future web-based interventions.

The second reminder at follow-up 1 and 2 in the Heart trial was made by telephone. To maximize responses, participants were offered to answer the questionnaire by pen and paper if they were reluctant to log in and answer via the portal. However, as this study focuses on use patterns, the questionnaires filled in by pen and paper answers were not considered. However, they have been reported in the Heart main study outcomes [8].

All data were logged using the portal. Researchers decided what to log beforehand but did not influence the data during data collection. There are some missing data, especially regarding reminders, and the data were not logged properly when the study commenced. However, the quality of the data extracted and analyzed in this study was high and reliable.

### Conclusions

Although use patterns differed slightly between the 2 studies, some general conclusions can be drawn. Most people who consented to participate in the study commenced by completing the baseline questionnaires within 1 week. Although many participants answered the questionnaires on the portal during office hours, approximately half of them did so during the weekend or in the evenings, suggesting that flexibility contributes to commencement and use. Participants in the study treatment groups tended to have lower completion rates for the follow-up questionnaires than those in the control or reference groups. This unexpected finding would be interesting for further investigations. Reminders were important to improve the completion rate of questionnaires at baseline and at follow-up. A second reminder was effective in increasing the completion rate. To summarize, our results show that log data provide a rich source of information for a better understanding of use patterns in web-based intervention and retention in eHealth trials. We found that commencement and retention are related to, among other things, flexibility, study design features, and reminders. Our results not only largely support previous findings but also indicate some unexpected user patterns to be investigated further. Refined logging and complementary interviews could potentially provide an even better understanding of these behavioral patterns. As we learn more about users’ detailed behaviors, we need improved intervention design and data collection that use the strengths and weaknesses of the internet format.

## Abbreviations

HADS: Hospital Anxiety and Depression Scale
iCBT: internet-based cognitive behavioral therapy
MADRS-S: Montgomery-Åsberg Depression Rating Scale-Self-Report
RCT: randomized controlled trial
